# CDK4/6 inhibitor: from quiescence to senescence

**DOI:** 10.18632/oncoscience.256

**Published:** 2015-10-20

**Authors:** Akihiro Yoshida, J. Alan Diehl

**Affiliations:** Department of Biochemistry and Molecular Biology, Hollings Cancer Center, The Medical University of South Carolina, SC, USA

**Keywords:** MDM2, ATRX, senescence, quiescent, CDK4/6

The CDK4 and CDK6 kinases (CDK4/6) are the first cyclin dependent kinases to be activated and initiate transition through G1 phase of the cell cycle. In response to mitogenic growth factors, the CDK4/6 kinase together with one of three D-type cyclins (D1, D2, D3) initiates G1 progression by virtue of its capacity to phosphorylate the retinoblastoma protein (RB), a bona fide tumor suppressor and Gate Keeper of cell division. Phosphorylation of RB in turn results in de-repression of E2F transcription factors thereby triggering expression of genes whose products drive S-phase entry and progression (1). Cyclin D1 dysregulation occurs in a majority of human cancers, a direct result of gene amplification or mutations that disrupt its protein degradation. CDK4 amplification or activating point mutations are also observed in select malignancies. The end result of such aberrations is elevated CDK4 catalytic function, increased cell division and decreased dependence on extracellular mitogenic growth factors for cell proliferation. These observations have contributed to significant efforts to develop selective small molecule CDK4/6 inhibitors with the hope that such entities would have significant anti-cancer benefit. PD0332991 (Palbociclib), a highly selective inhibitor of CDK4 (IC50: 0.011 μM) and CDK6 (IC50: 0.016 μM), has been shown to be highly efficacious in a variety of cell culture models with regard to its capacity to suppress cell cycle progression through inhibition of CDK4/6 kinase activity in an RB-dependent manner and it is currently being tested in clinical trials for malignancies such as mantle cell lymphoma, breast cancer, and colorectal cancer (2).

While acute inhibition of CDK4/6 is associated with reversible cell cycle withdraw or quiescence, some recent investigations have provided provocative evidence that Palbociclib treatment can in fact trigger irreversible withdraw, a state referred to as senescence (3, 4, 5). However the mechanisms that determine whether Palbociclib evokes quiescence versus senescence are yet to be established. In work described by Kovatcheva et al a new molecular mechanism wherein MDM2 and ATRX determine cell fate following CDK4/6 inhibition in cancer cells derived from several distinct cancer etiologies such as well-differentiated and dedifferentiated liposarcoma (WD/DDLS), lung cancer, and glioma (6). In this work, WD/DDLS cell lines were classified based upon cell fate following Palbociclib exposure: quiescence (non-responders) versus senescence (responders). While both groups had the expected reduction in RB phosphorylation, the responders also exhibited a significant decrease in MDM2 levels after prolonged CDK4 inhibition. Importantly, the phenotype is RB dependent, but is p53 and p16^INK4a^-independent. The capacity of MDM2 knock down to trigger senescence from quiescent phase in a combination with Palbociclib in non-responders, provides evidence that the reduction of MDM2 is in fact causative in the response and no simply an indirect consequence.

While the authors were unable to ascribe novel mechanistic insights with regard to MDM2 targets in the senescence response, additional experiments revealed that auto-ubiquitylation of MDM2 is essential for down regulation following Palbociclib exposure. Since MDM2 auto-ubiquitylation is regulated by HAUSP/USP7, a de-ubiquitylating enzyme, one might expect that its alteration of HAUSP/USP7 function might also impact senescence. Consistently, cell senescence could be induced by HAUSP/USP7 knockdown. However alteration of HAUSP/USP7 levels and association with MDM2 did not correlate with responder versus non-responder status demonstrating that HAUSP/USP7 does not contribute directly to determine cell fate following Palbociclib exposure.

Novel molecular insights into the cell fate switch arose from interrogation of a molecule that has been implicated in tumor cell escape from senescence; ATRX, ALT (Alternative lengthening of telomeres) associated protein. Critical analysis of ATRX in responders versus non-responders revealed differential post-translational modification of the c-terminus ATRX. The nature of this modification is currently unknown, nevertheless phosphorylation is a likely candidate modification. Knockdown of ATRX in responders rescued MDM2 loss and rendered these cells refractory to senescence but not quiescence revealing a functional link among ATRX, MDM2 and cell fate.

While the mechanistic insights provided in this work will provide a critical foundation for further investigations of the molecular mechanisms that underlie a cells decision to undergo the conversion of quiescence to senescence (geroconversion), their impact would be limited if these same observations were not of value in clinical responses. Importantly, Kovatcheva et al also evaluated their model in biopsies available in a clinical trial utilizing Palbociclib in WD/DDLS patients revealing that MDM2 loss following Palbociclib exposure correlated with response.

**Figure 1:**
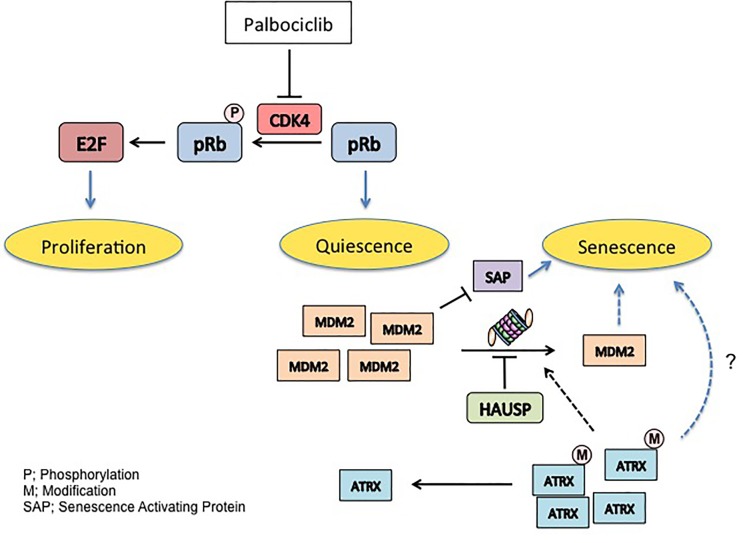
MDM2 and ATRX regulate geroconversion in response to Palbociclib

Palbociclib has been approved breakthrough therapy designation from FDA, however the molecular mechanisms underlying CDK4 inhibitor suppress tumor growth remain poorly define. Kovatcheva et al provide novel insights into the mechanisms that govern geroconversion. MDM2 and ATRX clearly contribute to cell fate following CDK4 inhibition in WD/DDLS and potentially other epithelial cancers (Figure 1). Ultimately, the insights provided in this work have distinct diagnostic implications and suggest the possibility that targeting MDM2 or ATRX by small molecule in a combination with CDK4 inhibitor might be promising for cancer therapy.
